# Improving the performance of true single molecule sequencing for ancient DNA

**DOI:** 10.1186/1471-2164-13-177

**Published:** 2012-05-10

**Authors:** Aurelien Ginolhac, Julia Vilstrup, Jesper Stenderup, Morten Rasmussen, Mathias Stiller, Beth Shapiro, Grant Zazula, Duane Froese, Kathleen E Steinmann, John F Thompson, Khaled AS AL-Rasheid, Thomas MP Gilbert, Eske Willerslev, Ludovic Orlando

**Affiliations:** 1 Centre for GeoGenetics, Natural History Museum of Denmark, Copenhagen University, 5-7 Øster Voldgade, København, 1350, Denmark; 2 Department of Biology, The Pennsylvania State University, 326 Mueller Lab, University Park, PA, 16802, USA; 3 Department of Tourism and Culture, Government of Yukon, Yukon, Palaeontology Program, PO Box 2703 L2A, Whitehorse, Yukon Territory Y1A, 2C6, Canada; 4 Department of Earth and Atmospheric Sciences, University of Alberta, Edmonton, Alberta T6G 2E3, Canada; 5 Applications, Methods and Collaborations, Helicos BioSciences, One Kendall Square Bldg 200LL, Cambridge, MA, 02139, USA; 6 Present address: Broad Institute of Massachusetts Institute of Technology and Harvard, Cambridge, MA, 021423, USA; 7 Present address: NABsys Inc, 60 Clifford Street, Providence, RI, 0290, USA; 8 Zoology Department, College of Science King Saud University, P.O. Box, 2455, Riyadh, 11451, Saudi Arabia

**Keywords:** Ancient DNA, True Single Molecule DNA Sequencing, Next-Generation Sequencing

## Abstract

**Background:**

Second-generation sequencing technologies have revolutionized our ability to recover genetic information from the past, allowing the characterization of the first complete genomes from past individuals and extinct species. Recently, third generation Helicos sequencing platforms, which perform true Single-Molecule DNA Sequencing (tSMS), have shown great potential for sequencing DNA molecules from Pleistocene fossils. Here, we aim at improving even further the performance of tSMS for ancient DNA by testing two novel tSMS template preparation methods for Pleistocene bone fossils, namely oligonucleotide spiking and treatment with DNA phosphatase.

**Results:**

We found that a significantly larger fraction of the horse genome could be covered following oligonucleotide spiking however not reproducibly and at the cost of extra post-sequencing filtering procedures and skewed %GC content. In contrast, we showed that treating ancient DNA extracts with DNA phosphatase improved the amount of endogenous sequence information recovered per sequencing channel by up to 3.3-fold, while still providing molecular signatures of endogenous ancient DNA damage, including cytosine deamination and fragmentation by depurination. Additionally, we confirmed the existence of molecular preservation niches in large bone crystals from which DNA could be preferentially extracted.

**Conclusions:**

We propose DNA phosphatase treatment as a mechanism to increase sequence coverage of ancient genomes when using Helicos tSMS as a sequencing platform. Together with mild denaturation temperatures that favor access to endogenous ancient templates over modern DNA contaminants, this simple preparation procedure can improve overall Helicos tSMS performance when damaged DNA templates are targeted.

## Background

Ancient DNA has been shown to survive in fossil material for over thousands to even hundreds of thousands of years [1-3]. However, *post-mortem* DNA damage reactions, which fragment the DNA backbone into short pieces and generate hydrolytic and oxidative base derivatives, often limit the amount of DNA templates preserved [4-6]. Together with DNA-DNA crosslinks [7,8] and by-products of the Maillard reaction [9], these modifications could limit the success of PCR amplification and thus the ability to incorporate ancient DNA fragments into DNA libraries, such as those required for sequencing on second-generation sequencing platforms [10]. As a result of such DNA degradation, the overwhelming majority of DNA within ancient DNA extracts often consists of exogenous contaminant DNA templates, mainly originating from environmental microbes [11], with endogenous DNA levels rarely greater than a few percent of the sequenceable templates [12,13].

Notable exceptions have been reported. For example, ancient keratinous materials such as hair, horn and nail, are relatively exempt from exogenous contamination [14,15], as is the occasional bone that has been exceptionally well preserved in both permafrost [16] and temperate environments [17].

Several different targeted re-sequencing techniques have been demonstrated to be able to preferentially access ancient DNA templates over exogenous contaminants, but these have been limited to the characterization of complete mitochondrial genomes [18,19], complete plasmids [20] and complete exomes [21]. The exact efficiency of such methods is also unclear with reported enrichment values ranging over several orders of magnitude and subject to whether or not clonal collapse is considered in the calculation [22]. So far, no DNA capture method has been successfully used for recovering gigabases of sequence information and complete genomes. As a result, the characterization of ancient genomes is still principally dependent on shotgun sequencing [17,23-25]. The introduction of methods that would improve the balance of endogenous over exogenous sequences obtained would therefore be especially beneficial in the emerging field of paleogenomics, since such methods would serve to improve sequencing depth and consequently the quality of the genome drafts generated [17,23,25]. Additionally, since most DNA extraction approaches are destructive, improving current methods would help to minimize damage to valuable samples, a point of critical importance in cases where the fossil record is limited (*e.g.* for Denisovans, a newly discovered group of archaic hominins, currently known by no more than three fossils).

Recently, Green and collaborators have shown that enzymatic restriction digestion can be used to successfully deplete the environmental microbe component within Illumina sequencing libraries, thus enriching the fraction of sequenced material of endogenous origin (in their case, Neandertal) [25]. Similarly, we have recently demonstrated that true Single Molecule DNA Sequencing (tSMS), as performed on the Helicos HeliScope platform, advantageously complemented Illumina GAIIx platforms when sequencing DNA extracted from a Pleistocene horse bone preserved in permafrost [10]. Indeed, a simple modification to the conventional tSMS DNA template preparation procedure, involving a modified denaturation temperature prior to template 3’ poly-A tailing, increased the efficiency of endogenous sequence recovery, suggesting that the efficiency of tSMS could be further improved for sequencing ancient DNA extracts [10]. Inspired by this observation, in this study, we further explore the potential of the tSMS procedure when sequencing ancient DNA extracts through two new modifications, namely oligonucleotide spiking and DNA phosphatase treatment. We show that both approaches increase tSMS performance, with up to 3.3-fold enrichment in the amount of endogenous sequence information recovered per sequencing channel. In addition, we confirm the existence of molecular preservation niches in bones [10,26], from which DNA can be preferentially extracted, leading to enriched fractions of endogenous DNA. Such niches should be preferentially extracted for characterizing the complete genomes of individuals and species from the past.

## Results and discussion

Two horse bone fossils preserved in permafrost were tSMS-sequenced on the Helicos platform using from 110 to 1100 FOV (fields of view) per channel. Overall sequence yield was expected to be directly proportional to the number of FOV. The first bone, CA, originated from Thistle Creek, Yukon Territory, Canada, and was associated with infinite radiocarbon dates (>50,300BP). The second bone, TP, was excavated in the Taymir Peninsula and was radiocarbon dated at 13,389 ± 52 BP. Each bone was extracted in duplicate, henceforth referred to as CA1 and CA2 for sample CA, and TP1 and TP2 for sample TP. In addition, for sample TP, the residual bone powder that remained undigested after the initial DNA extraction was subjected to redigestion until completely digested. DNA was subsequently purified from this second fraction, generating two additional extracts, referred to below as TP1RE and TP2RE.

### DNA denaturation temperatures

**Figure 1:**
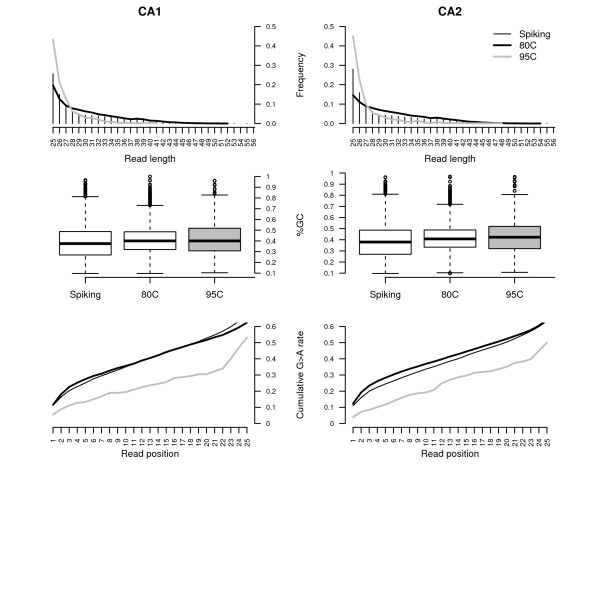
**Sequence features of the population of tSMS reads recovered from sample CA (>50,300 BP).** Sample CA was extracted in duplicate and both extracts were tSMS sequenced on a Helicos 1100 FOV channel following different template preparation procedures as described in the methods section. *Top:* Read length distribution (spiked sequences are reported as vertical bars). *Middle:* Read %GC contents. *Right:* Cumulative guanine to adenine misincorporation rates as a function of the distance from sequencing start. This class of mismatch derives from the *post-mortem* deamination of cytosine residues and can be taken as a proxy for *post-mortem* DNA damage.

Ancient DNA molecules are short, damaged, and showing overhangs at both termini. In contrast to the denaturation temperature at 95°C used in standard template preparation for the Helicos platform, mild denaturation temperatures limit further fragmentation of ancient DNA molecules [10]. For all extracts considered, when compared to a high denaturation temperature of 95°C, a more mild denaturation temperature of 80°C was found to yield: (1) a higher fraction of endogenous sequences (Tables 1 and 2); (2) longer reads (Figure 1, top); (3) lower overall %GC contents (Figure 1, middle), and; (4) higher levels of *post-mortem* cytosine deamination (Figure 1, bottom). Such enrichment could not be explained by the difference in volumes of CA extracts sequenced following denaturation at the respective temperatures since this would be expected to yield differences in the overall total number of sequences recovered but not in the relative proportions of the different fractions of reads present in the extracts. These observations are in agreement with previous observations derived from a more limited number of bone extracts [10]. This suggests that the presence of deaminated cytosine residues reduces the number of hydrogen bonds between DNA strands and thereby likely favor a preferential denaturation of endogenous ancient templates, especially compared to longer, hence more thermostable, exogenous templates. Interestingly, the younger sample (TP) exhibited lower levels of cytosine deamination than the older sample (CA; Additional file 1, Figure S1).

**Table 1:** tSMS sequencing of ancient DNA extracts from sample CA (>50,300BP)

**Sample**	**Platform**	**#**	**Conditions**	**#Seqs**	**nucDNA**	**bp**	**mtDNA**	**bp**	**%Endo.**
CA1	1100 V	1	Spiking	4,559,011*	57,946	1,728,020	3	107	1.27%
	1100 V	1	80°C	787,255	29,837	896,617	11	365	3.79%
	1100 V	1	95°C	35,055	1,020	27,242	0	0	2.91%
CA2	1100 V	1	Spiking	4,888,026*	54,266	1,599,775	3	90	1.11%
	1100 V	1	80°C	730,418	55,546	1,717,054	12	385	7.61%
	1100 V	1	95°C	51,550	1,231	33,023	0	0	2.39%

Sample CA was extracted in duplicate (CA1 and CA2), and identical volumes of the extracts were tSMS sequenced on a Helicos 1100 FOV platform following different template preparation procedures as described in the methods section. The total number of sequences without (#Seqs) and with (*) filtering for oligonucleotides used for spiking, as well as the number of hits mapping the horse reference nuclear (nucDNA) and mitochondrial (mtDNA) genome with mapping qualities higher than 25 but with no hit on the human reference genomes hg19 and rCRS are reported. The total sequence length covered by these reads is indicated in bp. The fraction of endogenous reads is estimated by summing the number of reads identified in nucDNA and mtDNA and dividing by the total number of reads generated per channel (minus reads corresponding to spiking oligonucleotides in the specific case of spiking experiments).

**Table 2:** tSMS sequencing of ancient DNA extracts from sample TP (13,389 ± 52BP)

**Sample**	**Platform**	**#**	**Conditions**	**#Seqs**	**nucDNA**	**bp**	**mtDNA**	**bp**	**%Endo.**
TP1	110 V	1	80°C, Phosphatase	1,980,210	85,738	2,885,229	53	1,845	4.33%
	110 V	1	80°C	234,094	28,797	998,007	8	255	12.30%
	110 V	1	95°C	228,871	12,573	438,111	5	162	5.50%
TP1RE	110 V	1	80°C, Phosphatase	1,607,848	161,284	5,597,277	99	3,378	10.04%
	550 V	8	80°C, Phosphatase	16,290,720	1,480,012	46,444,135	935	29,637	9.09%
	110 V	1	80°C	214,070	48,304	1,718,507	29	991	22.58%
	110 V	1	95°C	356,586	51,052	1,798,348	31	1,169	14.33%
TP2	110 V	1	80°C, Phosphatase	2,088,705	177,944	6,147,860	50	1,713	8.52%
	110 V	1	80°C	216,159	53,463	1,879,011	16	560	24.74%
	110 V	1	95°C	354,155	62,259	2,170,975	15	516	17.58%
TP2RE	110 V	1	80°C, Phosphatase	233,613	32,045	1,094,783	10	318	13.72%
	110 V	1	80°C	213,958	59,530	2,072,120	26	928	27.84%
	110 V	1	95°C	247,784	55,848	1,939,018	22	781	22.55%

Sample TP was extracted in duplicate, and identical volumes of the extracts were tSMS sequenced on Helicos 110 FOV channels following different template preparation procedures as described in the methods section. The total number of sequences (#Seqs) as well as the number of hits mapping the horse reference nuclear (nucDNA) and mitochondrial (mtDNA) genome with mapping qualities higher than 25 but with no hit on the human reference genomes hg19 and rCRS are reported. The total sequence length covered by these reads is indicated in bp. The fraction of endogenous reads is estimated by summing the number of reads identified in nucDNA and mtDNA and dividing by the total number of reads generated per channel.

### Molecular niches

**Figure 2:**
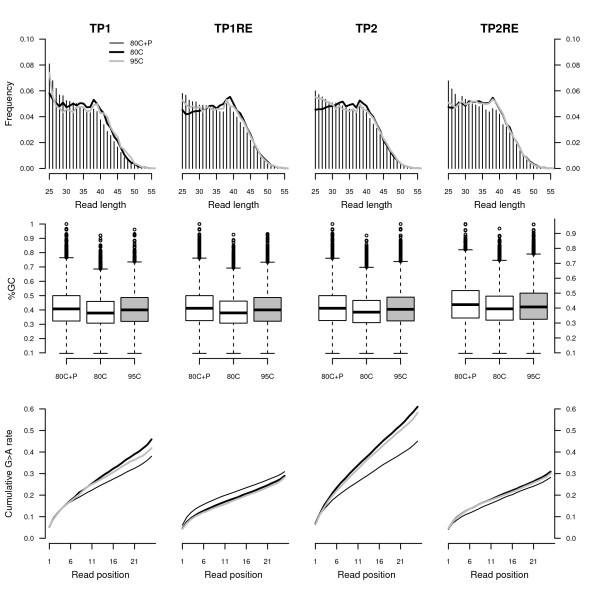
**Sequence features of the population of tSMS reads recovered from sample TP (13,389 ± 52 BP).** Sample TP was extracted in duplicate and identical volumes of the extracts were tSMS sequenced on a Helicos 110 FOV channels following different template preparation procedures as described in the methods section. The analyses are restricted to the fraction of reads identified as originating from the horse genome. *Top:* Read length distribution (spiked sequences are reported as vertical bars). *Middle:* Read GC contents. *Right:* Cumulative guanine to adenine misincorporation rates as a function of the distance from sequencing start. This class of mismatch derives from the *post-mortem* deamination of cytosine residues and can be taken as a proxy for *post-mortem* DNA damage.

The cytosine deamination levels observed in sequencing reads recovered from the extracts generated by redigesting residual pellet that was left-over from the first extraction (TP1RE and TP2RE), were found to be significantly lower than those observed in the sequencing reads generated from the initial extraction (TP1 and TP2) (Figure 2, bottom). In addition, re-extracted samples showed higher endogenous sequence yield regardless of the DNA denaturation temperature used during the template preparation procedure (Table 2). This confirmed our previous observations with other bone material [10]. We hypothesize that the initially undigested bone powder likely consisted of larger bone crystals, hence required additional time before full digestion could be completed. Crystal growth, and the related diagenetic increase in bone crystallinity observed *post-mortem*, could contribute to the entrapment and protection of endogenous DNA molecules in larger crystals, as observed with the TP1RE and TP2RE extracts. Conversely, an increase in bone porosity, and related fluid circulation, could result in microbial attack and the dissolution of crystals, which could potentiate the deposition of exogenous DNA templates on the fraction of bone powder that appeared easily decalcified (here, TP1 and TP2 extracts) [27,28]. We anticipate that the preferential extraction of larger bone crystals would offer a simple strategy for acquiring a higher proportion of endogenous DNA, thus improving the performance of shotgun sequencing.

### Oligonucleotide spiking

We additionally explored the potential of two other sequencing template preparation approaches, namely, oligonucleotide spiking and DNA phosphatase treatment, to improve the performance of tSMS on ancient DNA extracts. Such improvement could be either direct by increasing the relative ratio of endogenous content, or indirect, through higher total sequence recovery rates per channel.

In comparison to fresh material, degraded bone samples yield relatively low DNA concentrations that are up to several orders of magnitude below those observed in fresh bone extracts [29,30]. While samples with extremely low DNA yields are expected to perform poorly on any sequencing platform, such samples are still sequenceable on the Helicos platform when they are spiked with oligonucleotides. In this system, a camera scans the sequencing flow cell and records images of each field of view. After each nucleotide addition, the images must be aligned with each other in order to properly assign sequence information. If the DNA is at a very low concentration the images cannot be aligned properly, but the addition of spikes increases the total yield of DNA sequences, allowing more accurate image alignment so that it becomes possible to obtain sequences from extremely small numbers of endogenous DNA molecules [31]. This method has been shown to work effectively with modern DNA samples present at very low concentrations.

In order to test this idea on ancient DNA extracts, we focused on extracts CA1 and CA2. These extracts showed relatively low levels of endogenous DNA content (ca. 2.4% – 2.9% with 95°C as the denaturation temperature) and delivered low numbers of sequences per channel on a 1100 FOV platform (Table 1). For each extract, DNA templates prepared using 80°C and 95°C as denaturation temperatures were pooled, and spiked just prior to sequencing with a 40 pM solution consisting of 30 oligonucleotides just prior to sequencing (see Methods). The reads that derived from oligonucleotides were identified and filtered by mapping against those 30 known sequences. The results were then compared to the sequencing performance observed when no oligonucleotide spiking was performed. The fractions of extracts that were spiked with a mixture of exogenous oligonucleotides exhibited lower endogenous sequence ratios than unspiked extracts (1.11% – 1.27% *versus* 3.79% – 7.61%; Table 1). If the spiking procedure was equivalent to the standard procedure then a larger number of endogenous sequences would have been expected (see Methods). In particular, 142,076 – 143,299 and 179,788 – 181,159 horse reads would have been expected following spiking for extracts CA1 and CA2, respectively, in contrast to the 57,946 and 54,266 actually observed (Table 1). This difference shows that oligonucleotide spiking reduced our capability to access endogenous ancient reads. BLAST analyses revealed that the subset of sequence reads that was filtered for the sequence of oligonucleotide spikes presented higher proportions of unidentified sequences (97.6% and 97.8% for samples CA1 and CA2, respectively) compared to unspiked controls (88.1% and 93.3%). Likewise, we detected a 6.3-fold to 14.2-fold reduction in the proportion of BLAST hits assigned to bacteria following oligonucleotide spiking. This change in sequence composition suggests that a significant fraction of the reads consisted in oligonucleotide sequences that could not be filtered, possibly due to the presence of substantial levels of sequencing errors and/or the formation of chimeric sequencing reads.

**Figure 3:**
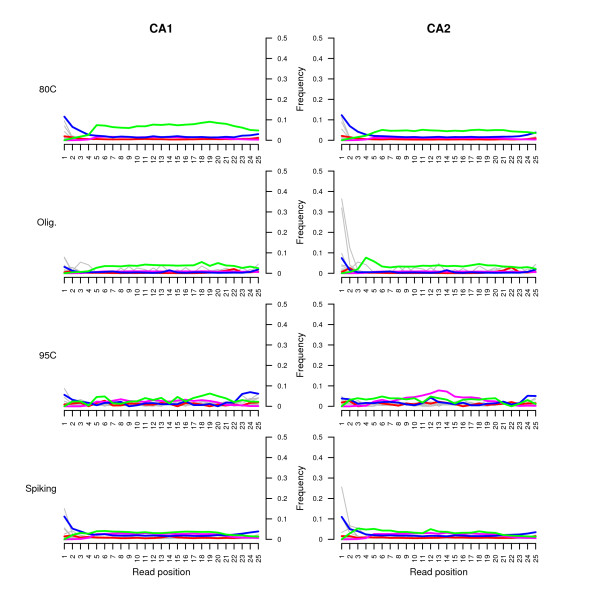
**Nucleotide misincorporation patterns observed on extracts CA1 and CA2.** The frequencies of all possible mismatches and indels observed between the horse genome and the reads are reported in grey as a function of the position on sequencing reads, except for C→T, G→A, insertions, and deletions that are reported in blue, red, pink, and green respectively. Only the first 25 nucleotides sequenced are considered. *Left:* CA1 extract. *Right:* CA2 extract. Template preparation procedures for tSMS sequencing are reported on the left hand side of the graphs. All graphs correspond to the analyses of horse reads (80°C, 95°C, and 80°C + Spiking) except for the ones labelled 80°C olig. that refer to the analysis of reads mapping against the 30 oligonucleotides used for spiking.

Further analyses of the sequences revealed that the population of horse DNA sequences recovered following oligonucleotide spiking exhibited a skewed %GC contents (Figure 1, middle). This lower %GC-content (average = 38.5%) might have resulted from the presence of unfiltered oligonucleotide sequences. Following oligonucleotide spiking, we found levels of cytosine deamination similar to the ones observed in the absence of spiking at a denaturation temperature of 80°C (Figure 1, bottom). The observed misincorporation pattern was in agreement with that expected for ancient DNA molecules, ie. an excess of G to A mismatches at the 5’-ends of reads (Figure 3, bottom).

Despite its ability to access genuine ancient sequence information, the oligonucleotide spiking approach tested here could not be recommended for sequencing ancient DNA extracts unless further developments are performed in order to optimize the filtering of oligonucleotide sequences and to prevent the shifts observed both in base composition and sequence diversity.

### DNA phosphatase treatment

A previous comparison of tSMS shotgun sequencing of modern *versus* ancient (Pleistocene horse bone) DNA extracts revealed no major difference in the base composition of the genomic region preceding sequencing reads [10]. In particular, blocking sites were found to be enriched in guanine residues due to the preferential incorporation of dGTP Virtual Terminator during the locking reaction (Additional file 1, Figure S2). Additionally, genomic coordinates located upstream of blocking sites were enriched for thymine residues most likely resulting from the preferential capture of poly-A rich genomic regions by oligo-dT probes linked to the surface of the flow cell [10] (Additional file 1, Figure S2). In contrast, Illumina sequencing of the same extracts has confirmed the model of DNA fragmentation through depurination presented by Briggs and colleagues, with excessive proportions of guanine and adenine residues at genomic positions preceding the sequencing start site [32].

The absence of a clear signal for depurination in tSMS reads has been proposed to result from the terminal transferase enzyme used for poly-A tailing in the Helicos template preparation procedure, which might show non-optimal activity on modified 3’-ends of depurinated sites [10]. After depurination the DNA backbone undergoes further cleavage at the site of base loss through β-elimination reactions, leaving DNA fragments with modified 3’-ends, which could be incompatible with poly-A tailing and further tSMS sequencing [4,33]. Phosphorylation is one of the many modifications possibly present at 3’-ends of ancient templates. Hence, we decided to test whether dephosphorylation of ancient DNA extracts could restore access to a significant fraction of ancient templates and therefore could improve the overall performance of tSMS by increasing either the overall number of sequences generated per channel or the fraction of endogenous sequences recovered. As mild denaturation temperatures had been shown to deliver higher endogenous sequence yields (see above), we selected an enzyme with 3’ DNA phosphatase activity that could be heat inactivated at 65°C. In addition, the enzymatic buffer was selected to be compatible with a further polyA-tailing reaction in order to avoid extra template purification steps. For each of the four extracts generated from sample TP a fraction was treated with DNA phosphatase and the template preparation procedure was performed using 80°C as the denaturation temperature. Controls were run using an identical template preparation procedure but with no phosphatase treatment.

In all but one duplicate (extract TP2RE) DNA phosphatase treatment generated a 7.5 – 9.7-fold enrichment in the overall number of sequences produced per sequencing channel (Table 2). This enrichment was paralleled by a 2.0 – 2.9-fold decrease in the ratio of endogenous sequences (Table 2), suggesting that exogenous templates benefited more from the DNA phosphatase treatment than endogenous DNA molecules. This was observed for the four independent experiments performed and could result from a saturation effect whereby DNA phosphatase restored all ancient molecules with the phosphorylated 3’-ends that were present in the relatively small volume of extracts analyzed (0.5 μl).

Except for extract TP2RE, DNA phosphatase treatment resulted in a higher coverage of the horse genome per channel, as the increase in the overall number of sequences outweighed the loss resulting from the decrease in relative endogenous content. In this way the DNA phosphatase treatment allowed us to retrieve 15.7 Mb of sequence information from four 110 FOV channels, while only 6.7 Mb could be recovered in controls with a similar DNA denaturation temperature but no DNA phosphatase treatment. One sample (TP2RE) showed, however, lower absolute sequence yield after phosphatase treatment, most likely due to a channel-specific drop in sequencing quality. Even though all 110 FOV channels were sequenced on the same run, a series of known factors can lead to channel-to-channel variation, including 1) inhomogeneous deposition of primers (causing DNA reads to be located too close to each other at one extreme or not enough reads per image at the other extreme); 2) air bubbles in the channel that cause inefficient distribution of reagents, and; 3) laser focusing issues caused by inhomogeneity of the flow cell surface. With the channel considered, the overall yield of aligned sequences was lower than all the other channels, phosphatased or not; additionally, the rates of endogenous sequences appears lower than non-phosphatased samples of the same type, in agreement with all the other phosphatased samples (Table 2). This channel-specific drop in sequencing quality has limited the number of sequences recovered, but has not changed misincorporation patterns, cumulative damage distributions and %GC content, that all appear similar to what observed for sample TP1RE.

**Figure 4:**
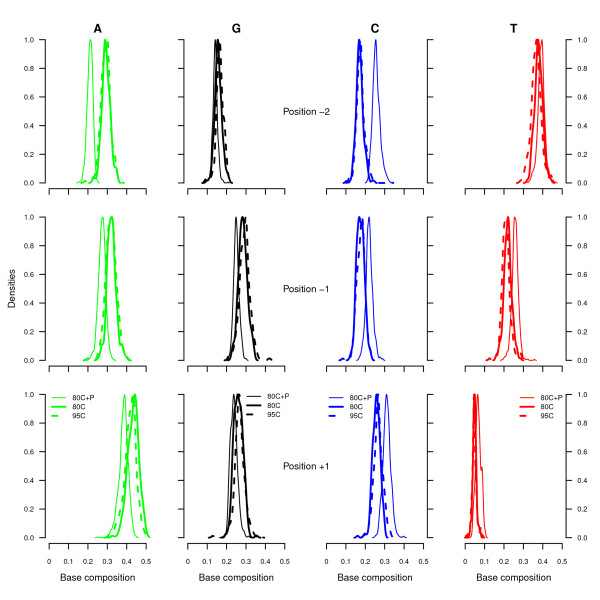
**Base composition at the 5’-end of tSMS reads and preceding genomic regions.** Read base composition is reported for the first position sequenced (position 1, bottom). In addition, the base composition of the genomic region is indicated for the two positions preceding the beginning of tSMS reads. The first (position −1, middle) corresponds to the locking site while the second (position −2, top) corresponds to the genomic position following the last nucleotide preserved in the ancient DNA template and located on the complementary strand (see Additional file 1, Figure S2).

Following DNA phosphatase treatment the base composition at the genomic position preceding the sequencing start was found to be enriched in pyrimidines (position −1; Figure 4, middle). This position corresponds to the blocking site, which was previously shown to preferentially consist of guanine residues [10]. In addition to affecting the base composition at the blocking site, the DNA phosphatase treatment was found to change the base composition at position −2 from the sequencing start, which corresponds to the position of the first nucleotide following the 3’-end of the ancient DNA strand (Additional file 1, Figure S2). After DNA phosphatase treatment this position was found to be enriched in cytosine residues, and depleted in equivalent proportions of adenine residues (Figure 4, top). As Helicos sequencing reads provide the sequence of the strand complementary to the ancient DNA template, this suggests that the first nucleotide following the 3’-end of the ancient DNA strand consisted of an excess of guanine residues, in agreement with the model of DNA fragmentation through depurination [34,35], and with previous analyses using Illumina sequencing that have reported a preferential enrichment in guanine residues at the genomic coordinate preceding the beginning of sequencing reads [10]. Hence, the DNA phosphatase treatment restored the signal of DNA depurination which was nearly absent from previous tSMS analyses of ancient DNA templates, confirming that modification of 3’-ends, in particular phosphorylation, precluded efficient poly-A tailing of ancient DNA templates with terminal transferase.

Furthermore, we noted that, following DNA phosphatase treatment, the base composition of the first nucleotide sequenced was enriched in cytosine residues (Figure 4, bottom). This enrichment occurred at the expense of adenine residues. Overall, horse reads recovered from the DNA phosphatase treatment were found to be slightly shorter than reads recovered without DNA phosphatase treatment (Figure 2, top) and the base composition of these reads was similar to those recovered following the use of a 95°C denaturation temperature (Figure 2, middle). The fraction of horse reads recovered after treatment of TP1 and TP2 extracts with DNA phosphatase exhibited G→A cumulative misincorporation rates that were lower than those observed without DNA phosphatase treatment, suggesting that DNA phosphatase allowed preferential access to ancient DNA templates with lower levels of cytosine deamination (Figure 2, bottom). More specifically, G→A cumulative misincorporation rates ranged from 38.1% – 45.1% in the first 25 nucleotides sequenced following DNA phosphatase treatment in contrast to 45.9% – 61.0% in the absence of this treatment. No significant difference in overall G→A cumulative misincorporation rates were found in horse sequences recovered from extracts TP1RE and TP2RE, despite different DNA denaturation temperatures and/or DNA phosphatase treatment.

We should caution that DNA phosphatase treatments of ancient DNA extracts could be deleterious if second-generation sequencing methods and not tSMS were used as sequencing approaches, since the former require building ancient templates into DNA libraries through adapter ligation at phosphorylated 5’-ends. This has been demonstrated by Briggs et al. [35] who have shown that the fraction of mammoth reads was drastically reduced following incubation with Calf Intestinal Alkaline Phosphatase in the absence of further rephosphorylation of 5’ ends with PNK. Conversely, treatment with DNA phosphatase increased the amount of templates accessible to DNA polymerases used for filling-in adapter sequences by restoring 3’ hydroxyl termini. Therefore the pre-treatment of ancient DNA extracts with DNA phosphatase before library building, followed by a proper end repair step rephosphorylating 5’ ends, could be used for improving our ability to insert ancient DNA fragments into DNA libraries and consequently for improving the performance of 454 and Illumina sequencing of ancient DNA templates. The exact benefit of this approach for second-generation sequencing, however, remains to be determined. We anticipate that it will prove highly dependent on the relative efficiency of Antarctic phosphatase over T4 Polynucleotide Kinase, as the latter is part of the standard end-repair enzymatic cocktail used for library building and could also catalyse the removal of 3’-phosphoryl groups from polynucleotides.

### Mitogenome analysis

Overall, we have demonstrated that sequencing of DNA purified from redigested bone powder, and the use of DNA template preparation procedures combining a mild DNA denaturation temperature and DNA phosphatase treatment, significantly increased the amount of endogenous sequences retrieved from ancient bones using tSMS. Following these findings, we decided to perform further tSMS sequencing on the TP1RE extract using our modified DNA template preparation procedure (Table 2). A total of eight channels were used on a 550 FOV platform, generating 46.4 Mb of horse DNA sequences. When added to the information recovered from the twelve 110 FOV channels this yielded 75.2 Mb of horse DNA sequence for the 13ky-old sample (sample TP). A total of 42,253 nucleotides could be aligned with no ambiguity on the horse mitochondrial genome, corresponding to a global coverage of 2.53X (Accession Nb. NC_001640).

**Figure 5:**
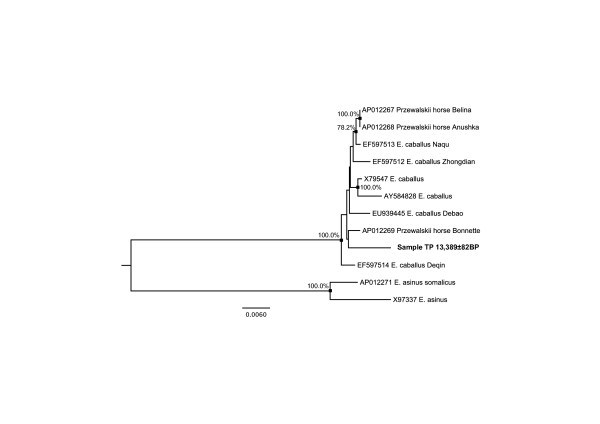
**Phylogenetic relationships among ancient and modern horses.** All sequences were downloaded from GenBank, except for sample TP that has been analyzed for the first time in this study. Accession numbers are reported together with available information about the different samples considered. The analysis was restricted to sites showing a minimal coverage depth of 2 and >50% of sequence identity among reads (11,316 sites). Phylogenetic inference is performed with Maximum Likelihood using a HKY + Γ 8 model of molecular evolution including a gamma correction for rate heterogeneity among sites. Node supports are estimated from a total number of 1,000 bootstrap pseudo-replicates and are reported for those above 75%.

We subsequently used the mitochondrial information to assess further the quality of tSMS sequences. We first focused on nucleotide positions (sites) covered with a depth of 2 and calculated the proportion of sites showing identical bases among reads as a conservative estimate of the quality of the data generated by tSMS on ancient DNA templates. In addition, we further considered sites showing a minimal sequencing depth of 3 and calculated the proportion of these with >50% base agreement among reads. Ignoring indels, we found identical sequences in 97.8% of the sites covered at a depth of 2. Furthermore, levels of accuracy ranging from 99.97% – 100.0% were found at sequence depths equal to or greater than 3 (a total of 7,510 sites), suggesting that a strict consensus approach considering sites with minimal sequencing depth of 2 should deliver high quality mitochondrial data. We consequently built a Maximum Likelihood phylogenetic tree using such sites (11,316 in total) together with donkey and horse mitogenomic data available from the literature [36-40]. As expected, the late Pleistocene mitogenome sequence data recovered from sample TP was found to cluster among horse sequences with maximal bootstrap support (Figure 5). Interestingly, one haplotype characterized from the wild Przewalski’s horse exhibited maximum sequence similarity to the late Pleistocene sequence. However, the latter grouping was not supported by high bootstrap values, but is in agreement with the notion of this haplotype originating from a Przewaski’s horse specimen that is thought to have no recent domestic horse genetic contribution to its known pedigree [40].

## Conclusions

In this study we have confirmed that large bone crystals that resist a first digestion step in EDTA-rich buffers provide access to molecular preservation niches in ancient bones, resulting in high endogenous sequence contents as well as lower levels of cytosine deamination. Pending further confirmation for a wider range of preservation conditions, such niches could be targeted as a means of optimizing the characterization of complete ancient genome sequences through shotgun sequencing. This further shows that non-destructive extraction methods consisting of smooth digestion in decalcifying buffers do not provide access to the optimal fraction of DNA templates preserved in bone specimens. In addition, we show that even simple temperature shifts and/or enzymatic treatments, have a major impact on the population of molecules retrieved from tSMS. Overall, this suggests that the amount of ancient DNA sequences that are accessible using current methods could potentially be increased by improving extraction procedures and restoring accessibility to 3’ termini. We therefore suggest that DNA from large bone crystals should be preferentially extracted, and when tSMS is used as a sequencing method, DNA templates should be prepared following DNA phosphatase treatment and using mild denaturation temperatures. Spiking of ancient DNA extracts with oligonucleotides of known sequences also appears to be a promising means to increase tSMS yields further.

## Methods

### DNA extraction

The DNA extract used for testing the extent to which oligonucleotide spiking could improve the performance of tSMS was prepared from a horse fossil bone excavated at Thistle Creek in Pleistocene permafrost deposits. The sample consists of two pieces of bone (CA1 and CA2) collected from a larger bone (TC21 YG148.2) that has already been successfully analyzed by tSMS and that has been associated with an infinite radiocarbon date (OxA-23933 >50,300BP; and UBA-16493 and UBA-17013 >50,505BP) [10]. The DNA phosphatase experiments were performed on DNA extracts from a humerus bone fragment from another horse fossil (CGG10023 XA08-53) that has been preserved in the permafrost (Taymir peninsula) and that has been associated with a finite radiocarbon date (UBA-16479 13,389 ± 52BP). All fossil bones were extracted using ancient DNA facilities at the Centre for GeoGenetics, Copenhagen, Denmark. Samples were drilled to powder at low speed and this bone powder was then extracted at 37°C under agitation using a 48 hours digestion in 5 ml of EDTA buffer (EDTA 0.5 M, pH = 8.0; N-lauryl Sarcosyl 0.5%; Proteinase K 1 mg/ml). A total of 0.45 and 0.46 grams of bone powder were digested for samples CA1 and CA2, whereas the digestion was replicated twice on 4.0 and 2.2 grams of bone powder for sample CGG10023. The CGG10023 sample derived from the initial digestion of 4.0 grams of bone powder will be referred to in the main text as TP1 and that from the 2.2 grams sample as TP2. The supernatants of these first digestions, recovered after spinning the digestion solution at 2,000 rpm for 5 minutes, were transferred to 30 kDa Amicon centrifugal filter units (Millipore) and spun at 3,000 rpm for a minimum of 30 minutes. A volume of ca. 200 μl was recovered from each Centricon column and further concentrated to 150 μl in an EB buffer using Minelute columns (QIAgen) following the manufacturer’s instructions except that the elution was performed 15 minutes after loading the EB buffer on the column and incubating the column at 37°C. The bone pellets that were left undigested after this first extraction of TP1 and TP2 were further extracted by adding 5 ml of EDTA buffer and following the same procedure as above. These re-extractions will be referred to TP1RE and TP2RE in the main text.

### tSMS sequencing

Helicos tSMS reactions were performed at Helicos BioSciences Corporation facilities in Cambridge, Massachusetts, USA. For samples CA1 and CA2, we followed the same methodology as described in [10] using 8 μl of DNA extract and two different DNA denaturation temperatures while preparing DNA templates for tSMS (80°C and 95°C, respectively). Two microliters of the 95°C extracts and 4 μl of the extracts denatured at 80°C were loaded per channel on a 1100 FOV sequencing platform using a total number of four channels (Table 1). In addition, 6 μl of the CA1 95°C extract and 4 μl of the CA1 80°C extract were pooled, spiked with polyA-tailed oligonucleotides (final concentration 40 pM) and loaded on one channel of the 1100 FOV platform. A similar procedure was followed for the CA2 extracts. This procedure aimed at ensuring that there were enough DNA molecules being sequenced to provide good image registration.

Since different volumes of the 80°C and 95°C denatured extracts were pooled, we estimated the expected minimal endogenous ratio Rmin for spiking experiments using a weighted average of the endogenous ratios observed without spiking. For each spiking experiment, we further assumed a binomial distribution with Rmin as the probability of success for estimating the number of expected endogenous sequences given the total number of sequences generated. Five percent and 95% quantiles were estimated using the function qbinom in R [41].

The effects of different denaturation temperatures and of DNA phosphatase treatment were tested on the four extracts recovered from sample CGG10023 (TP1, TP1RE, TP2 and TP2RE) using 110 FOV and 550 FOV. For the phosphatase reactions, 0.5 μl of the DNA extracts were mixed with 8 μl of nuclease-free water, 1 μl of NEB Antarctic Phosphatase 10X Reaction buffer and 2.5 units of NEB Antarctic Phosphatase. Reactions were incubated for 30 minutes at 37°C. Reactions were stopped by inactivating the enzyme at 65°C for 15 minutes. For the poly-A tailing reaction, the heat-inactivated phosphatase reactions were mixed with 0.8 μl of nuclease-free water, 2 μl of NEB Terminal Transferase 10X buffer and 2 μl of a 2.5 mM CoCl_2_ solution. This mixture was heated at 80°C for 5 minutes in a thermocycler for denaturation. For DNA extracts that were not phosphatased, 0.5 μl of the DNA extracts were mixed with 10.3 μl of nuclease-free water, 2 μl of NEB Terminal Transferase 10X buffer and 2 μl of a 2.5 mM CoCl_2_ solution. This was then heated at 80°C or 95°C for 5 minutes for denaturation. All the extracts were rapidly cooled on ice to minimize re-annealing of the denatured DNA strands. For all poly-A tailing reactions the volume of the previous mixes was increased to 20 μl through the addition of 5 units of NEB Terminal Transferase, NEB BSA (to 1X final concentration) and dATP at a final concentration of 10 μM in the 20 μl reaction. The single stranded DNA molecules were poly-A tailed for 1 hr at 37°C. Reactions were stopped by inactivating the enzyme at 70°C for 10 minutes. As the DNA is prone to re-annealing during the tailing step, heating at 80°C or 95°C followed by rapid cooling was repeated before 10 μl of 3’-end blocking mastermix (NEB Terminal Transferase buffer, 250 μM CoCl_2_, 5U NEB Terminal Transferase, 10 μM ddATP) was added to the tailing reaction volume. The 3’-end blocking reactions were performed for 1 hr at 37°C and stopped by denaturing the enzyme at 70°C for 20 minutes. DNA that may have reannealed during the blocking reaction was converted back to single strands by repeating the previous heating-rapid cooling conditions prior to loading the samples on the flowcell. 20 μl of sample consisting of 1 μl of the tailed and blocked DNA reaction, 9 μl of nuclease-free water and 10 μl of 2X hybridization buffer was added to each channel and allowed to hybridize for 1 hr at 37°C. The buffer was then rinsed away and the extra bases of the poly-A tailed reaction were filled in with dTTP and then locked in place with the first non-TTP base [42]. Sequencing was carried out using Virtual Terminator^©^ Nucleotides as described in [43]. The resulting sequence reads were then filtered for minimal sizes of 25 nucleotides and for artifactual sequences that closely matched the order of nucleotide addition. Sequence reads starting with a minimum number of 2 Ts were further trimmed as the latter could arise from incomplete fill and lock reactions. The remaining set of filtered reads was then analyzed.

### Sequence analysis

The DNA sequence data analyzed in this study are available on the NCBI Sequence Read Archive (SRA Accession Number SRA045862). Helicos reads were mapped using BWA against two equid mitochondrial genomes (Accession numbers: NC_001640 for the horse, and NC_001788 for the donkey), the human reference genome (hg19) and the horse reference genome (equCab2, filtered for the mitochondrial genome and chromosome Un), each available at the UCSC Genome Bioinformatic website: http://genome.ucsc.edu/). Seed was disallowed for mapping using the –l option and providing a value larger than the size of the longest sequence reads. In addition, as tSMS shows high error rates, especially indels [43,44], gaps were allowed all along the reads, including at termini, by setting the -i option to zero [45]. The BWA output was further converted to sam format with the samse command and filtered for mapping quality scores higher than 25 using the view command in samtools. Reads mapping uniquely to the horse reference genome but showing no hit to the human reference were retained. This conservative approach was selected in order to filter for possible contamination with human DNA and remove spurious hits that could result from short sequencing reads. BLAST analyses of sequences obtained for samples CA1 and CA2, after filtering of spiking oligonucleotides for spiking experiments were performed using blastn [46] against the NCBI non-redundant database with the following options: -F F –e 0.01 –q −2, i.e., no masking for repeat regions, expect value threshold 0.01 and mismatch penalty adjusted to −2. Base compositions, fragmentation and nucleotide misincorporation patterns were generated using SAM files as input and the map Damage package [47]. %GC contents and cumulative damage rates were plotted using mapDamage outputs and in-house-made R scripts [41].

Maximum Likelihood phylogenetic inferences were performed using PhyML 3.0 [48] and the robustness of nodes was estimated from 1,000 bootstrap pseudo-replicates. The best-fit model of substitution (HKY + G8) was selected in jModeltest [49] using the Akaike Information Criterion (AICc). The ML phylogeny was plotted using FigTree v1.3.1 available at http://tree.bio.ed.ac.uk/software/figtree.

## Abbreviations

tSMS, True Single Molecule DNA Sequencing; aDNA, Ancient DNA; NGS, Next-Generation Sequencing; FOV, Field of View.

## Competing interests

None.

## Authors’ contributions

LO and JT designed research. LO, JV and JS extracted ancient DNA. LO and AG analyzed tSMS data. LO and JV performed phylogenetic analyses. DF, GZ, BS, MS contributed fossils. KS and JT performed tSMS. KAH, KS, JT, TG, EW contributed methods, reagents and tools, and valuable input for data interpretation. LO wrote the manuscript. All authors read and approved the final manuscript.

## Supplementary Material

Additional file 1: **Figure S1.** Contrasting nucleotide misincorporation patterns from sample CA (>50,300 BP) and sample TP (13,389±52 BP). The frequencies of all possible mismatches and indels observed between the horse genome and the sequencing reads are reported in grey as a function of their position on the sequencing reads, except for C→T, G→A, insertions, and deletions that are reported in blue, red, pink, and green respectively. Only the first 25 nucleotides (left) or the last 25 nucleotides (right) sequenced are considered. Figure S2. Helicos tSMS sequencing: basics and coordinate definition. Helicos tSMS is not dependent on library building and amplification but rather performs direct sequencing of single molecules following a simple template preparation procedure consisting of DNA denaturation and poly-A tailing (A_n_, red) of single stranded templates with terminal deoxynucleotidyl transferase. Poly-A tailed templates are further captured with oligo-dT-50 probes linked to the surface of a flow cell. Fill-in (T, black) and lock (B, blue) steps are designed to fill any remaining nucleotide 3’ of the probe and to avoid sequencing the region complementary to the poly-A tail. Assuming 100%-efficiency for the locking step, the first nucleotide sequenced with tSMS corresponds to a position complementary to the penultimate nucleotide of the preserved ancient DNA strand. This position is referred to as position +1 in Figure 4. The last nucleotide of the ancient DNA template corresponds to the locking site; this position is referred to as position −1 in Figure 4. The first genomic position following the end of the ancient DNA strand is referred to as position -2. In Figure 4, the base composition at positions +1, -1 and −2 is given according to the strand sequenced by tSMS (see red arrows). Base composition at positions −1 and −2 are retrieved according to the base composition at the corresponding genomic coordinates, while for position +1, the base composition is estimated directly from the read sequence information. Click here for file
