# Supplementary material for: Improving the performance of true single molecule sequencing for ancient DNA
Source: BMC Genomics. 2012 May 10;13:177. doi: 10.1186/1471-2164-13-177 (PMC3430569; doi:10.1186/1471-2164-13-177)
Supplement: Additional file 1: — Figure S1. Contrasting nucleotide misincorporation patterns from sample CA (>50,300 BP) and sample TP (13,389±52 BP). The frequencies of all possible mismatches and indels observed between the horse genome and the sequencing reads are reported in grey as a function of their position on the sequencing reads, except for C→T, G→A, insertions, and deletions that are reported in blue, red, pink, and green respectively. Only the first 25 nucleotides (left) or the last 25 nucleotides (right) sequenced are considered. Figure S2. Helicos tSMS sequencing: basics and coordinate definition. Helicos tSMS is not dependent on library building and amplification but rather performs direct sequencing of single molecules following a simple template preparation procedure consisting of DNA denaturation and poly-A tailing (An, red) of single stranded templates with terminal deoxynucleotidyl transferase. Poly-A tailed templates are further captured with oligo-dT-50 probes linked to the surface of a flow cell. Fill-in (T, black) and lock (B, blue) steps are designed to fill any remaining nucleotide 3’ of the probe and to avoid sequencing the region complementary to the poly-A tail. Assuming 100%-efficiency for the locking step, the first nucleotide sequenced with tSMS corresponds to a position complementary to the penultimate nucleotide of the preserved ancient DNA strand. This position is referred to as position +1 in Figure 4. The last nucleotide of the ancient DNA template corresponds to the locking site; this position is referred to as position −1 in Figure 4. The first genomic position following the end of the ancient DNA strand is referred to as position -2. In Figure 4, the base composition at positions +1, -1 and −2 is given according to the strand sequenced by tSMS (see red arrows). Base composition at positions −1 and −2 are retrieved according to the base composition at the corresponding genomic coordinates, while for position +1, the base composition is estimated directly from [file 1471-2164-13-177-S1.doc]

**Improving the performance of True Single Molecule Sequencing for ancient DNA**

Aurelien Ginolhac, Julia Vilstrup, Jesper Stenderup, Morten Rasmussen, Mathias Stiller, Beth Shapiro, Grant Zazula, Duane Froese, Kathleen E. Steinmann, John F. Thompson, Khaled A.S. AL-Rasheid, Tom Gilbert, Eske Willerslev, Ludovic Orlando^*^.

* Corresponding author

**Additional Data File**

|  |
| --- |
| **Supplemental Figure 1. Contrasting nucleotide misincorporation patterns from sample CA (>50,300BP) and sample TP (13,389±52BP).** |

| 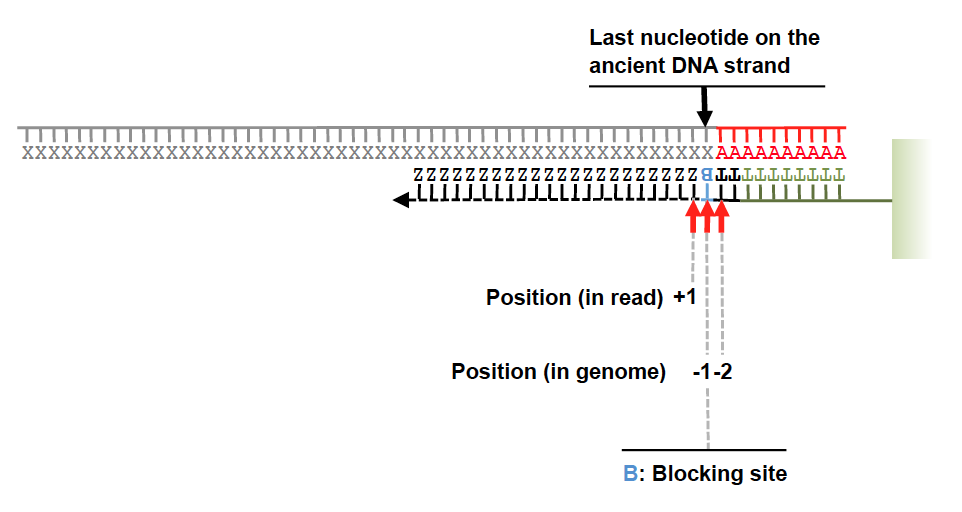 |
| --- |
| **Supplemental Figure 2. Helicos tSMS sequencing: basics and coordinate definition.** |
